# Supplementary material for: HIVprotI: an integrated web based platform for prediction and design of HIV proteins inhibitors
Source: J Cheminform. 2018 Mar 9;10:12. doi: 10.1186/s13321-018-0266-y (PMC5845081; doi:10.1186/s13321-018-0266-y)
Supplement: Supplementary file 2 — Additional file 2. Source code of HIVProtI web server. [file 13321_2018_266_MOESM2_ESM.zip › HIVprotI_Source-code/hivprotI/weka-3-6-6/doc/weka/associations/package-tree.html]

weka.associations Class Hierarchy


---


|  |  |  |
| --- | --- | --- |
| **Overview** \| **Package** \| Class \| **Tree** \| **Deprecated** \| **Index** \| **Help** \| **Weka's home** | |  |
| PREV   **NEXT** | **FRAMES**    **NO FRAMES**     **All Classes** |  |


---


## Hierarchy For Package weka.associations

**Package Hierarchies:**: All Packages

---

## Class Hierarchy

- java.lang.Object
  - weka.associations.**AbstractAssociator** (implements weka.associations.Associator, weka.core.CapabilitiesHandler, java.lang.Cloneable, weka.core.RevisionHandler, java.io.Serializable)
    - weka.associations.**Apriori** (implements weka.associations.CARuleMiner, weka.core.OptionHandler, weka.core.TechnicalInformationHandler)- weka.associations.**FPGrowth** (implements weka.core.OptionHandler, weka.core.TechnicalInformationHandler)- weka.associations.**GeneralizedSequentialPatterns** (implements weka.core.OptionHandler, weka.core.TechnicalInformationHandler)- weka.associations.**PredictiveApriori** (implements weka.associations.CARuleMiner, weka.core.OptionHandler, weka.core.TechnicalInformationHandler)- weka.associations.**SingleAssociatorEnhancer** (implements weka.core.OptionHandler)
              - weka.associations.**FilteredAssociator**
              - weka.associations.**Tertius** (implements weka.core.OptionHandler, java.lang.Runnable, weka.core.TechnicalInformationHandler)
    - weka.associations.**AssociatorEvaluation** (implements weka.core.RevisionHandler)- weka.core.**Check** (implements weka.core.OptionHandler, weka.core.RevisionHandler)
        - weka.core.**CheckScheme**
          - weka.associations.**CheckAssociator** (implements weka.core.RevisionHandler)
        - weka.associations.**FPGrowth.AssociationRule** (implements java.lang.Comparable<T>, java.io.Serializable)- weka.associations.**FPGrowth.BinaryItem** (implements java.lang.Comparable<T>, java.io.Serializable)- weka.associations.**ItemSet** (implements weka.core.RevisionHandler, java.io.Serializable)
              - weka.associations.**AprioriItemSet** (implements weka.core.RevisionHandler, java.io.Serializable)- weka.associations.**LabeledItemSet** (implements weka.core.RevisionHandler, java.io.Serializable)
              - weka.associations.**Messages**- weka.associations.**PriorEstimation** (implements weka.core.RevisionHandler, java.io.Serializable)- weka.associations.**RuleGeneration** (implements weka.core.RevisionHandler, java.io.Serializable)
                    - weka.associations.**CaRuleGeneration** (implements weka.core.RevisionHandler, java.io.Serializable)
                    - weka.associations.**RuleItem** (implements java.lang.Comparable<T>, weka.core.RevisionHandler, java.io.Serializable)

## Interface Hierarchy

- weka.associations.**Associator**- weka.core.**OptionHandler**
    - weka.associations.**CARuleMiner**

## Enum Hierarchy

- java.lang.Object
  - java.lang.Enum<E> (implements java.lang.Comparable<T>, java.io.Serializable)
    - weka.associations.**FPGrowth.AssociationRule.METRIC\_TYPE**

---


|  |  |  |
| --- | --- | --- |
| **Overview** \| **Package** \| Class \| **Tree** \| **Deprecated** \| **Index** \| **Help** \| **Weka's home** | |  |
| PREV   **NEXT** | **FRAMES**    **NO FRAMES**     **All Classes** |  |


---
